# How ARDS should be treated

**DOI:** 10.1186/s13054-016-1268-7

**Published:** 2016-04-06

**Authors:** Luciano Gattinoni, Michael Quintel

**Affiliations:** Department of Anesthesiology, Emergency and Intensive Care Medicine, University of Göttingen, Robert-Koch-Straβe 40, 37075 Göttingen, Germany

## Abstract

The Berlin definition criteria applied at positive end-expiratory pressure (PEEP) 5 cm H_2_O reasonably predict lung edema and recruitabilty. To maintain viable gas exchange, the mechanical ventilation becomes progressively more risky going from mild to severe acute respiratory distress syndrome (ARDS). Tidal volume, driving pressure, flow, and respiratory rate have been identified as causes of ventilation-induced lung injury. Taken together, they represent the mechanical power applied to the lung parenchyma. In an inhomogeneous lung, stress risers locally increase the applied mechanical power. Increasing lung homogeneity by PEEP and prone position decreases the harm of mechanical ventilation, particularly in severe ARDS.

Various etiologies can induce an inflammatory process in the lung parenchyma. In some patients, the inflammation spreads throughout the entire lung, leading to the diffuse edema that defines the acute respiratory distress syndrome (ARDS) [1]. The dependent lung regions tend to collapse under the increased lung weight [2], and only non-dependent lung regions remain open for ventilation. Gasless regions and reduced lung size are the anatomical basis of the two main symptoms of ARDS: oxygen refractory—fraction of inspired oxygen (FiO_2_)-resistant—hypoxemia [3] and decreased lung compliance [4].

As a result, several measures must be undertaken simultaneously. Treatment aimed at correcting the etiology depends strictly on the underlying disease causing the ARDS. Several less specific treatments that target the pathogenesis, such as steroids [5], statins [6], and a variety of anti-mediators, have been proposed and tested for their ability to contain or prevent the spread of the inflammatory process. Unfortunately, none of them has shown a clear-cut positive effect on outcome. On the other hand, the symptomatic treatment of gas exchange is totally unspecific and independent of the cause of ARDS because its goal (that is, maintaining appropriate blood gas tensions) and its risks hinge on the same factor: the edema and its extent.

Accordingly, there are two first steps that must be undertaken in patients with ARDS: diagnosis, from which we derive the specific treatment, and determination of ARDS severity [7, 8]. To assess the severity, we must, ideally, quantify the edema by computed tomography scan [9] or other imaging techniques [10] or by determining the amount of extravascular lung water [11]. In practice, the Berlin [7] classification assessed at 5 cm H_2_O of positive end-expiratory pressure (PEEP) is a reasonable estimate of the extent of edema and of lung recruitability, which increases from mild to severe ARDS [12]. We strongly recommend this approach, as it allows one to choose the most rational, and consequently less hazardous, respiratory support regimen in any given patient.

Mechanical ventilation does not cure ARDS but simply buys time by maintaining a gas exchange sufficient for survival. This benefit is provided by taking over the function of the respiratory muscles. In patients with ARDS, the respiratory muscles are unable, for several reasons, to provide sufficient power to move gas in and out of the lungs. The effects of mechanical ventilation on oxygenation are twofold: they allow the precise titration of FiO_2_ in the delivered gas, and they provide sufficient pressure during the inspiratory phase to open some of the collapsed pulmonary units, thus allowing blood passing through these regions during inspiration to be oxygenated. But these units will collapse again during the expiratory phase if the PEEP is not sufficient [13–15]. Consequently, the effects of tidal ventilation alone on oxygenation are limited unless applied together with an appropriate PEEP level. Ventilation, on the other hand, is essential for carbon dioxide (CO_2_) elimination. In ARDS, the increased respiratory drive [16, 17] and the increased pulmonary dead space [18] increase the necessary minute ventilation to a level that is far greater than normal even if some degree of hypercapnia were to be accepted [19]. Indeed, ventilation of the “baby lung” implies the use of stress (driving pressure) and strain (tidal volume) [20] that is excessive for the dimensions of the residual ventilated lung. This problem obviously increases with the severity of ARDS. Therefore, although the risk factors associated with improving oxygenation are the use of high FiO_2_ and the opening and closing of lung units during the respiratory cycle [21, 22], the greatest risks of mechanical ventilation are associated with the necessity of eliminating CO_2_. In fact, depending on the severity of ARDS, the mechanical stress imposed on the “baby lung” may be such as to alter the extracellular matrix and thereby trigger further inflammation [23].

The damage associated with mechanical ventilation has been collectively labeled ventilator-induced lung injury, although the more realistic designation would be ventilation-induced lung injury (VILI), since it may occur even during spontaneous breathing [24]. VILI has been variously attributed to excessive tidal volumes [25], driving pressures [26], respiratory rates [27], and gas flows [28]. We believe that a unifying hypothesis should consider VILI to be the result of excessive mechanical power (that is, energy per unit time) applied to the lung tissue [29, 30], where “excessive” is relative to the “baby lung” dimensions. In addition, as pointed out by Mead et al. [31], if the mechanical power is distributed in an inhomogeneous lung, the tidal energy can be multiplied locally by the presence of stress risers [32, 33].

Accordingly, we believe that respiratory treatment should consist in minimizing, as much as possible, the applied mechanical power [29, 30] and the inhomogeneity of the lung [31, 32]. The mechanical power in this case is primarily the product of tidal volume, driving pressure [26], and respiratory rate [27, 33, 34]. One should note that PEEP itself does not produce any tidal energy load, as the delta volume is zero, except when first introduced [35]. Therefore, whatever maneuver reduces the applied mechanical power (such as reducing tidal volume), driving pressure or respiratory rate will reduce the probability of VILI. The disappointing results of high-frequency oscillation studies [36, 37] can be considered under the aspect of power: even small tidal excursions, multiplied by the driving pressure and by the hundreds of cycles per minute, may generate an intolerable mechanical load. For a given mechanical load, the risk of VILI decreases if the lung is made more homogeneous, thereby reducing the presence of stress risers [31, 32]. Two measures may increase lung homogeneity: an appropriate level of PEEP and prone positioning [38]. PEEP increases the homogeneity by preventing intertidal collapse [21, 22] and keeping the recruited pulmonary units open [14, 15]. The prone position increases lung homogeneity by counteracting the gravitational forces with a more favorable matching of lung to chest wall shape [38]. Both prone position and PEEP, however, produce their benefit only in patients with intermediate–severe and severe ARDS [39], in whom the high degree of lung recruitability [40] provides the anatomical basis for PEEP and the prone position to be effective.

## Conclusions

We do believe that the principles of ARDS treatment should be based on the following: diagnosis and specific etiological treatment and the classification of ARDS severity [7, 39] at a PEEP of 5 cm H_2_O [12]. In mild ARDS, mechanical ventilation does not cause problems. With increasing severity, the mechanical power applied to the lungs should be reduced as much as possible [29, 30], and a higher PEEP and prone position should be employed. In some patients, safe mechanical ventilation may not be possible. The identification of a reasonable power threshold for VILI would be the ideal parameter for the rational indication of extracorporeal lung support.

## Abbreviations

ARDS: acute respiratory distress syndrome
CO_2_: carbon dioxide
FiO_2_: fraction of inspired oxygen
PEEP: positive end-expiratory pressure
VILI: ventilation-induced lung injury
